# Analysis of multimorbidity compression using a latent variable in a mixed mixture model

**DOI:** 10.1186/s12963-025-00421-w

**Published:** 2025-10-24

**Authors:** Angela Andreella, Lorenzo Monasta, Stefano Campostrini

**Affiliations:** 1 Institute for Maternal and Child Health, IRCCS Burlo Garofolo, Trieste, Italy; 2 Department of Economics, Ca’ Foscari University of Venice, Venice, Italy

**Keywords:** Multimorbidity compression, Global burden of disease project, Latent trait model, Surveillance system PASSI

## Abstract

**Background:**

Multimorbidity, i.e., the co-presence of multiple diseases in an individual, is an increasing concern, particularly as the population ages. Addressing it is critical to improving health status and optimizing healthcare resources. Particularly relevant in this scenario is the concept of multimorbidity compression, i.e., the onset of chronic diseases is delayed more rapidly than the increase in life expectancy. According to this theory, the duration individuals spend in poor health should be shortened. Existing studies have started examining multimorbidity trends, yet often overlook the cumulative burden of multiple diseases.

**Methods:**

We define the multimorbidity concept as a latent variable estimated with the disease burden described by the disability weights from the Global Burden of Diseases (GBD) project. Using a mixed-mixture model, we analyze the nonlinear relationship between multimorbidity and socioeconomic traits, accounting for zero inflation and spatial variability in Italy. We use twelve years of the surveillance system PASSI data to investigate the multimorbidity compression concept.

**Results:**

Our findings suggest multimorbidity compression is acting in Italy: severe multimorbidities are increasingly concentrated later in life, indicating a positive impact of healthcare improvements on the quality of life. The phenomenon is observed in both socially advantaged and disadvantaged subpopulations.

## Introduction

Multimorbidity, i.e., the simultaneous occurrence of multiple diseases in an individual, is a growing concern, particularly in aging and chronically ill populations [1, 2]. It complicates diagnosis, treatment strategies, and overall well-being, often leading to reduced quality of life, higher mortality rates, and increased healthcare costs. Recognizing and adequately assessing multimorbidity is crucial for improving health status and optimizing resource allocation in healthcare systems [3, 4]. As populations age, understanding and addressing the complexities associated with multiple chronic conditions becomes even more urgent [5, 6].

To effectively address the challenges of an aging population, the most viable approach is through the lens of morbidity compression, which focuses on preventing behavioral risk factors such as smoking, poor diet, alcohol consumption, and physical inactivity [7]. Introduced in 1980 by Fries [8], this theory argues that it is possible not only to delay the onset of chronic diseases more rapidly than the extension of life expectancy, but also to reduce their incidence over time. As a result, the period during which individuals suffer from severe health conditions becomes shorter [9, 10]. This concept contrasts with the “Failures of Success” model, which suggests that medical advances prolong life but increase the time spent in poor health [11]. The notion of morbidity compression, primarily through the prevention of behavioral risk factors, has gained traction as one of the few viable approaches to maintaining the health of aging populations.

Despite growing interest [12–15], the morbidity compression concept remains mainly under-explored in Italy and in high-income countries [16]. Recent studies have begun to address this issue in Italy, yet limitations persist. For instance, Pastore and coauthors [7] defined multimorbidity based on the presence of at least one chronic disease, neglecting the cumulative impact of multiple coexisting conditions on the individual quality of life. They suggest that morbidity compression has occurred in Italy over the past decade, evident in two key trends: a delay in the average onset of chronic diseases and a reduction in the prevalence of having at least one chronic disease among pre-elderly individuals (ages 40–69). Notably, morbidity compression appears more pronounced among men and Italians with higher socioeconomic status.

In another study, Stival and coauthors [17] took a more granular approach by examining each chronic disease separately and focusing on geographical variability within the older population. They found no clear-cut answer: the presence and intensity of compression vary significantly depending on the types of diseases analyzed and the geographical area, i.e., Italian regions and/or local health authorities (Azienda Sanitaria Locale, ASL). For instance, cardiovascular diseases generally show compression at the national level. At the same time, tumor-related morbidity has increased in some areas, i.e., the northern regions and Sardinia, with Liguria and Emilia-Romagna experiencing the most significant rises.

Both studies analyzed data from the Italian PASSI surveillance system [18], as in this work. In contrast, other research, e.g., [19], used the National Health Interview Surveys (NHIS) data (https://www.istat.it/en/non-categorizzato/european-health-interview-survey-ehis/), conducted by the Italian National Institute of Statistics (ISTAT) every five years. Demeru and Egidi [19] indicate potential improvements in health conditions among Italy’s elderly population. Specifically, they found that the number of years spent without functional limitations increased more than the number of years lived with limitations. However, their analysis did not account for socioeconomic differences, which limits a nuanced understanding of how health trends vary across different population subgroups.

Another study not based on the PASSI data is the one from Moretti et al. [20], which analyzed the longitudinal European Union Statistics on Income and Living Conditions survey for Italy (IT-SILC, https://www.istat.it/informazioni-sulla-rilevazione/eu-silc/). They found evidence of disability compression among individuals aged 50–79 years. Here, compression was assessed through three indicators: an increase in disability-free life expectancy (DFLE), a simultaneous decrease in life expectancy with disability (DLE), and an increase in the H indicator, defined as the proportion of disability-free years (DFLE) over total life expectancy (LE) in this age range, across the study periods.

As said before, when analyzing morbidity compression in Europe and high-income countries, there is limited literature available. This scarcity is mainly due to several factors. The simultaneous investigation of morbidity and mortality is rarely feasible with the available data. Moreover, studies examining morbidity trends over extended periods are few and typically focus on a limited number of diseases. Finally, longitudinal cohort studies are mostly predictive in nature and often based on relatively small initial populations. When refreshment samples are not introduced, attrition leads to selective samples over time, which limits the ability to adequately study compression or expansion of multimorbidity [16].

However, some evidence of health improvements has been observed in Germany [21], where an increase was found in the proportion of 50–70-year-old men and women rating their health as “good” or “very good” between 1995 and 2015. This improvement is at least partly attributable to increased physical activity, consistent with Fries’s compression hypothesis [9]. Sacerdote et al. (2012) [22] found evidence of morbidity compression related to cardiovascular disorders in high-income countries. Similarly, Malki et al. (2014) [23] observed decreases in myocardial infarction and ischemic stroke incidence over time among men, while rates remained stable for women, analyzing a cohort of nearly three million Swedish residents born between 1932 and 1960, followed from 1987 to 2010. In the same vein, Aparicio et al. (2019) [24] reported a decline in ischemic stroke incidence among participants of the Framingham Heart Study.

Similarly, Payne et al. (2022) [25] analyze how patterns of health, morbidity, and disability have evolved across successive generations of older adults in the United States. Their study reveals that while self-rated health is improving at older ages (70+), there is little compression of disability, with life expectancy lived with chronic morbidities expanding significantly. Moreover, substantial disparities exist by education level and race/ethnicity, with disadvantaged subgroups experiencing increasingly disabled and unhealthy life expectancies. However, Crimmins and Beltrán-Sánchez (2011) [12] review evidence from the United States suggesting that morbidity compression is not occurring, especially when measured objectively by mobility loss or diagnosed diseases. This international literature informs our approach and underscores the importance of studying multimorbidity compression in the Italian context.

Our study addresses these gaps by considering multimorbidity as a whole, incorporating socioeconomic characteristics, and analyzing sub-populations defined by different behavioral risk factors.

The aim of this study can be summarized in three main questions: (1) Is the compression of multimorbidity occurring in Italy? (2) If so, is it happening uniformly across subpopulations defined by various socioeconomic characteristics? (3) When present, is multimorbidity compression primarily driven by improvements in behavioral risk factors, or are other mechanisms at play?

To address these questions, we firstly need to define a proper multimorbidity index. We then introduce a latent model that estimates the concept of multimorbidity using the disability weights [26] from the Global Burden of Disease (GBD) project [27, 28], incorporating the methodology of Andreella and coauthors [29]. As their work, we use the data from the Italian health surveillance system PASSI [18] to estimate the concept of multimorbidity in Italy. However, their analysis focused solely on the year 2019; our research examines twelve years of PASSI data. In addition, the latent model allows for a multimorbidity index with continuous support, unlike the index proposed by Andreella and coauthors [29], which has semi-continuous support. The continuous support of the latent index enables the capture of fine-grained variation in the underlying morbidity burden. In addition, using a statistical model also enables analysis of the impact of individual diseases on the construction of the latent index. By estimating the loadings of each observed indicator, the model quantifies the differential contribution of individual diseases to the latent construct, allowing for a more data-driven and structurally consistent representation of multimorbidity across individuals and subpopulations.

In our study, we then adopt a reflective approach to construct the multimorbidity index, treating non-communicable diseases (NCDs) as observable expressions of a single underlying condition. Unlike formative models–which assume that each condition contributes independently to the construct–this perspective recognizes and leverages the interdependence among indicators. The latent trait thus emerges from the observed patterns of co-occurrence, offering a more integrated and statistically coherent measure of multimorbidity. This framework also accommodates the limitations of our dataset: adding or removing an individual indicator does not significantly alter the conceptual foundation of the index. Rather than relying on predefined weights, the structure of the multimorbidity construct is shaped by the data itself, aligning closely with the inferential and descriptive goals of our analysis.

After estimating the concept of multimorbidity, we are able then to address our research questions defined above. We then fitted a mixed-mixture model to analyze the presence of multimorbidity compression within subpopulations characterized by different levels of socioeconomic status. The mixed-mixture model examines the relationship between socio-demographic variables and the multimorbidity index, accounting for both temporal (time of interview) and spatial (NUTS 2 level) factors.

Building on the three main research questions outlined above, we acknowledge that the cross-sectional nature of the data constrains our analysis. Therefore, to address question (1), we examine the effect of the survey year within the mixed-mixture model framework. Question (2) is explored by analyzing differences in the estimated multimorbidity index across subpopulations defined by various socioeconomic characteristics. Finally, to respond to question (3), we stratify the population by risk levels based on the presence or absence of behavioral risk factors, such as smoking, alcohol consumption, poor nutrition, and physical inactivity

Following prior research (e.g., [30–33]), we hypothesize that the compression of multimorbidity is occurring in Italy, but with varying intensity across socioeconomic groups. While the overall burden of multimorbidity may be decreasing, the rate of reduction might not be sufficient to equalize health outcomes across subpopulations. Socioeconomic characteristics [34] are therefore included in the analysis as potential effect modifiers of this process. Moreover, as behavioral risk factors such as smoking and alcohol consumption have been declining generally in Italy [35, 36], we examine whether these trends are the primary drivers of compression or whether other structural or medical improvements also play a significant role.

The manuscript is structured as follows. Section "Italian surveillance system PASSI data" presents the data analyzed from the cross-sectional Italian health surveillance system PASSI [18]. Section "Multimorbidity index" details the statistical approach employed to estimate the multimorbidity index using a weighted latent trait model, along with the corresponding results. Section "Ordered beta regression with random effect" discusses the mixed-mixture model utilized to investigate the three research questions. Finally, Section "Discussion" is dedicated to discussion.

## Italian surveillance system PASSI data

Our data stems from the Italian health surveillance system PASSI, a cross-sectional study to collect data about lifestyles and behavioral risk factors linked to the prevalence of NCDs. PASSI targets Italian adults aged 18 to 69 years. For more insight, refer to [18]. Additional information is available on the following webpage: https://www.epicentro.iss.it/passi/en/english.

The survey includes a question where respondents self-report a diagnosis of the five following NCDs: diabetes, kidney failure, bronchitis/emphysema/respiratory failure/, myocardial infarction/cardiac ischemia/coronary artery disease/, and tumors (including leukemias and lymphomas). We also examine socioeconomic variables, including sex (female/male), age (ranging from 18 to 69), educational level (classified as low if below high school and high otherwise), and economic status (assumed high if individuals can easily make ends meet by the end of the month, and low otherwise). Additionally, we analyze the year of the interviewer (from 2008 to 2019) and the spatial location (NUTS 2 level composed by nineteen regions: Abruzzo, Basilicata, Calabria, Campania, Emilia-Romagna, Friuli Venezia Giulia, Lazio, Liguria, Marche, Molise, Piemonte, Puglia, Sardegna, Sicilia, Toscana, Trentino-Alto Adige/Südtirol, Umbria, Valle d’Aosta/Vallée d’Aoste, and Veneto). Data from the Lombardia region are not included due to insufficient participation in the surveillance system.

While this study does not focus on the elderly population (65+), examining the age group of 18 to 69 is essential, as early life factors significantly influence long-term health status and the potential for “successful aging” [7]. In this perspective, we interpret changes in the distribution of multimorbidity as early indicators of morbidity compression. Although the clinical consequences of multimorbidity are typically more pronounced in older adults, evidence suggests that shifts in disease onset and accumulation begin earlier in life. Our analysis, therefore, captures the initial stages of these shifts, offering insights into potential compression dynamics before the traditional threshold of old age. Nevertheless, we acknowledge that definitive conclusions about morbidity compression would require follow-up in older populations.

**Fig. 1:**
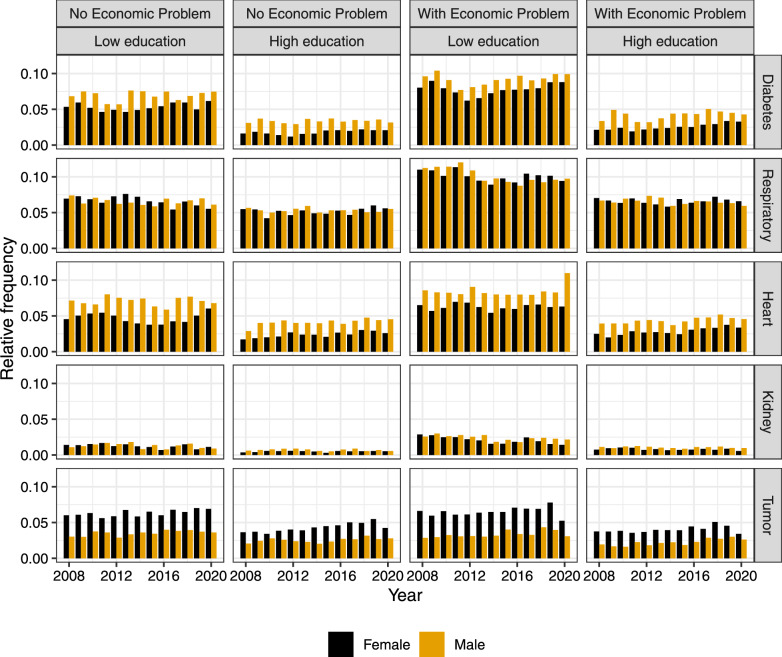
Relative frequency of each NCD by survey year, disaggregated by educational level (high vs. low), economic status (with vs. without economic difficulties), and sex (female vs male)

Finally, the dataset includes $53.18\%$ female respondents, $60.17\%$ of individuals with higher education, and $52.77\%$ of participants reporting economic difficulties. In total, there are 445,709 observations. Descriptive analyses focusing on the year 2019 are proposed by Andreella and coauthors [29] while Fig. 1 displays the relative marginal frequency of five NCDs by survey year, educational level, economic status, and sex.

## Multimorbidity index

Before addressing the three research questions introduced in Section "Introduction", we first define an appropriate index of multimorbidity. To this end, we rely on a latent trait model, which provides a continuous measure of morbidity burden from the observed set of chronic conditions. In particular, Subsection "Weighted latent trait model" outlines the statistical framework behind the proposed index, while Subsection "Results" presents the corresponding empirical results based on the PASSI data described in Section "Italian surveillance system PASSI data".

In Subsection "Results", we also report the item information function, which illustrates how each disease contributes to the measurement of the latent trait. This analysis, as well as the interpretation of the estimated coefficients in the latent trait model, is not strictly required for constructing the multimorbidity index or for the compression analysis, which remains our primary objective. However, they highlight a key advantage of the latent trait approach over the multiplicative index proposed by Andreella et al. [29].

### Weighted latent trait model

Fries and coauthors [9] emphasize that a measure of morbidity should focus on a broad form of disability, often using functional disability as a proxy. Reliably assessed by patient self-report, this metric aligns with patient perceptions of ill-health [9]. Following this perspective, we conceptualize multimorbidity as a latent variable based on the concurrent presence of the five NCDs detected by PASSI. To enrich our definition of multimorbidity in terms of functional disability and its impact on quality of life, we integrate the index proposed by Andreella and coauthors [29], which is grounded on two factors. The first is the perceived health variable, an ordinal categorical variable that takes values between 1 and 5, where 1 means excellent self-reported health and 5 means very bad self-reported health. The second is the disability weights [26] estimated from the GBD project [27, 28]. These disability weights offer a quantifiable measure of health loss associated with specific health conditions, allowing for a more standardized assessment of the burden imposed by various NCDs. Disability weight plays a crucial role in assessing the amount of time lost to health due to living with a specific disease condition [37].

Therefore, our approach responds to the critique by Fries and colleagues [9], who point out that simply counting chronic conditions does not fully reflect the true burden of morbidity. Diagnostic labels vary widely in severity and often do not correspond well to actual functional limitations or how people feel about their health. As Fries [9] emphasizes, functional disability is a reliable and sensitive measure of morbidity, easily assessed through patient self-report and closely linked to overall health and physical functioning [21, 38–41]. This justifies our use of a latent multimorbidity index that combines self-reported diagnoses with subjective health measures, better reflecting the concept and evidence behind morbidity compression.

In short, the multimorbidity index proposed by Andreella and coauthors [29] is essentially a composite disability weight. For each identified disease, the approach assigns a specific disability weight [26]. Given the extensive range of disability weights available for each NCD, Andreella and coauthors [29] employ a multi-step methodology to determine the most appropriate weight. The first step involves selecting a set of GBD weights for each disease identified in the PASSI data using text mining techniques. For each disease identified in the PASSI data, Andreella and coauthors [29] generate a set of keywords, including the name of the NCD and its synonyms. These keywords were then matched to the health state names used in the GBD project. Further details can be found in [29, Tables 1, 2]. Subsequently, the second step incorporates the perceived health variable from PASSI to link a single disability weight to each detected disease. Briefly, when the perceived health is rated between 1 and 3, the minimum disability weight is used. For a rating of 4, the average of the selected disability weights is employed, and for a rating of 5, the maximum disability weight is considered. If a respondent $j \in \{1, \dots , N\}$ reports multiple NCDs, resulting in multiple disability weights, these weights are combined using the following formula:

1 $$\begin{aligned} D_j = 1 - \prod _{i = 1}^{5}(1 - W_{ij}). \end{aligned}$$

Here, $W_{ij}$ represents the disability weight associated with NCD *i* in subject *j*, as determined by the abovementioned multi-step approach. We then refer to the multiplicative approach proposed by Andreella and coauthors [29]. Some empirical studies in the literature favor the multiplicative approach [42], which the GBD also utilized to combine disability weights in their analyses from 2010 and 2013 [43, 44]. The quantity $D_j$ represents the “cumulative disability weight”, summarizing the overall burden associated with the presence of multiple NCDs for individual *j*.

The primary limitation of the approach proposed by Andreella and coauthors [29] lies in the semi-continuous nature of the multimorbidity index, which complicates its analysis using statistical models that assume continuous support for the dependent variable. In addition, determining the individual impact of each disease in defining multimorbidity is not straightforward.

To address these issues, we conceptualize the multimorbidity index as a latent variable defined by the five NCDs identified in the PASSI data. We rely on the Latent Trait Model (LTM) [45], which permits, unlike the approach proposed by Andreella and coauthors [29], to explore the disease properties, such as the impact of each disease in a multimorbidity context, taking into account the measurement error. By modeling both the latent trait and the relationship between the observed variables and the latent trait, the LTM reduces the impact of measurement error. In an LTM, the observed variables (such as the presence or absence of diseases) are considered imperfect measures of the underlying latent trait (in our case, the multimorbidity concept).

Let $X_i$ denote the manifest variables with $i \in \{1, \dots , 5\}$, i.e., the five NCDs detected by PASSI. $X_{ij} \in \{0,1\}$ is distributed as a Bernoulli random variable with conditional expected value equals $\mathbb {E}(X_{ij} \mid z_j)=\pi _i(z_j) = \Pr (X_{ij} = 1 \mid z_j)$ for each individual $j \in \{1, \dots , N\}$. The latent trait model [45] is then defined as:

$$\begin{aligned} \text {logit}\{\pi _i(z_j)\} = \beta _{0i} + \beta _{1i} z_j \end{aligned}$$

where $\beta _{0i}$ can be interpreted as the prevalence of disease *i* (in particular $\text {expit}(\beta _{0i})$ corresponds to the probability that an individual with average multimorbidity ($z=0$) presents the condition) and $\beta _{1i}$ the effect of multimorbidity $\textbf{z} = [z_1, \dots , z_N]^\top$ on the log of the odds of having the disease *i*. Thus, the latent variable $z_j$ captures the concept of multimorbidity in subject *j*. Consistent with standard practice, the latent variable $z_j$ was assumed to follow a standard normal distribution. As a robustness check, we also estimated the model using a semi-parametric Davidson curve with 10 parameters [46]. The resulting AIC, BIC, and model fit statistics were virtually identical to those obtained under the normality assumption, indicating that our findings are not sensitive to the specific distributional form of $z_j$.

In the likelihood-based estimation process of the parameters $\beta _{0i}$, $\beta _{1i}$ and $z_j$, the observations $j = 1,\dots , N$ are weighted as follows:

2 $$\begin{aligned} w_j = \dfrac{(D_j + 1) N}{\sum _{j=1}^{N} D_j +N} \end{aligned}$$

where $D_j$ is the multimorbidiy index defined in Equation (1). Therefore, the observations are weighted considering the magnitude of health loss linked with specific health conditions.

The algorithm implemented follows Bock et al. (1988) [47], which employs a Gauss-Hermite quadrature approximation of the marginal distribution within the Expectation-Maximization (EM) framework. This method is available through the mirt package in R [48].

### Results

**Table 1:** Estimate values of the latent trait model’s parameters

**Disease**	$-\hat{\varvec{\beta }}_0$	$\hat{\varvec{\beta }}_1$
**Diabetes**	3.571	1.340
**Kidney failure**	6.082	2.077
**Respiratory**	2.880	0.974
**Heart**	3.924	1.647
**Tumor**	3.198	0.840

**Fig. 2:**
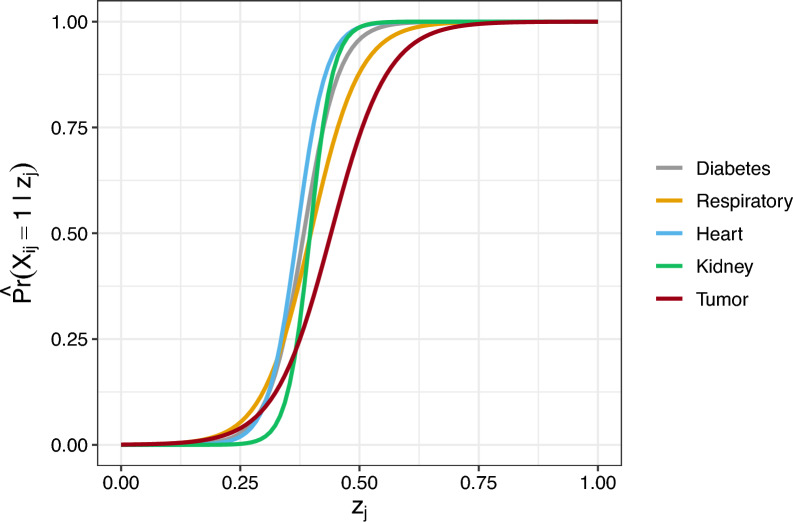
Estimated probabilities to have the diseases $i = 1, \dots , 5$ across several multimorbidity values

Table 1 shows the estimated values of the $\varvec{\beta }_0$ and $\varvec{\beta }_1$ vector of coefficient parameters for each NCD detected by PASSI. Figure 2 represents the estimated probability of having the *i* disease across several $z_j$ (i.e., multimorbidity) values.

We can note that the least prevalent NCD is kidney failure, which is also the most impactful one in terms of morbidity. In contrast, tumors are the most prevalent (along with respiratory disease) and the least impactful. One possible explanation is that kidney problems pave the way for the onset of various disabling diseases. Conversely, cancers, while widespread, exhibit a spectrum of impact, ranging from mildly disabling to significantly disabling. However, a mortality effect often influences the latter category (i.e., selecting respondents).

Another interesting measure that we can extract from an LTM is the “item information function” [49], which provides insight into how much a disease contributes to estimating the latent trait at different levels of $z_j$. The item information function is defined as

$$\begin{aligned} I_i(z_j) = \beta _{1i}^2 \Pr (X_{ij}=1 \mid z_j)(1-\Pr (X_{ij}=1 \mid z_j)). \end{aligned}$$

**Fig. 3:**
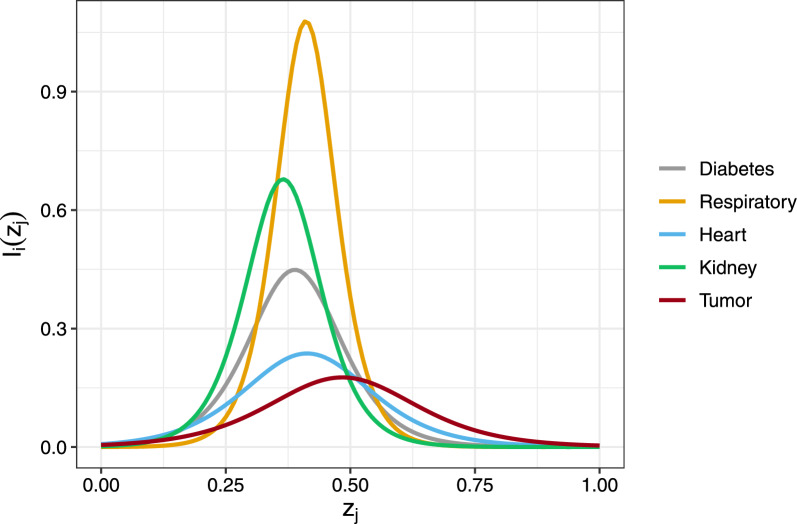
Disease information across different levels of multimorbidity $z_j$

These values are represented in Figure 3, where on the *y*-axis we have the information values and on the *x*-axis the multimorbidity values. We can note that respiratory disease and kidney failure are the most informative diseases when we have a multimorbidity around 0.35, i.e., low-medium level. However, tumors give information when a high level of multimorbidity is considered.

Thanks to these insights provided by the latent trait model, we can conclude, for example, that tumors, while highly prevalent, play a complex role in multimorbidity: they may not strongly drive increases in the overall burden but are crucial for capturing variability across both moderate and severe multimorbidity levels. To address the conditional independence assumption underlying the latent trait model, we conducted both global and item-level fit assessments. Overall model fit was evaluated using the $M_2$ statistic ($M_2 =209.44$, $\text {df} = 5$, $p < 0.001$), which, despite being significant—likely due to the large sample size—is accompanied by excellent approximate fit indices: $\text {RMSEA} = 0.01$ ($90\%\,\, \text {CI}: 0.009--0.0113$), $\text {SRMSR} = 0.017$, $\text {TLI} = 0.987$, and $\text {CFI} = 0.993$. These values indicate a good fit of the unidimensional model and suggest that the latent trait adequately captures the common structure across the observed indicators. In addition, we examined item-level fit statistics to detect potential local dependencies. While some items showed significant $S_{X^2}$ statistics (e.g., respiratory, cancer, and diabetes), the corresponding RMSEA values remained small ($\le 0.024$), indicating only modest deviations from local independence. Given the clinical plausibility of comorbid associations and the complexity of real-world data, some degree of residual correlation is expected. Nevertheless, the overall model performance suggests that the assumption of conditional independence is reasonably well met for latent trait estimation.

We report the results without using the weights defined in Equation (2) in section "Unweighted latent trait model".

## Ordered beta regression with random effect

After estimating the multimorbidity index, we set up a model for analyzing morbidity compression in socio-demographic subpopulations. This model is designed to capture important features, such as the variability among regions and the unique distribution characteristics of the multimorbidity index. Since the multimorbidity index has a zero-inflated distribution, likely due to a high proportion of healthy individuals, we adopt a mixed-mixture model framework to effectively handle the excess of zeros and the continuous range between zero and one.

Let $\textbf{y} = (y_1, \dots , y_j, \dots , y_N)^\top$ denote the vector of multimorbidity indices measured on the *N* respondents which were rescaled to take values in [0, 1], i.e.,

$$\begin{aligned} y_{j} = \dfrac{z_j - z_{(1)}}{z_{(N)} - z_{(1)}} \end{aligned}$$

where $z_j$ is the original multimorbidity score for individual *j* estimated by the LTM defined in Section "Multimorbidity index" and $z_{(1)}$ and $z_{(N)}$ are the minimum and maximum values of $\textbf{z}$, respectively. We emphasize that this transformation is performed only after model estimation; it does not affect the LTM results but simply rescales the latent scores to the [0, 1] interval. This choice enhances interpretability of the multimorbidity index, as values close to 0 naturally correspond to the absence of comorbidity—a crucial feature given the zero-inflated distribution—while higher values indicate increasing comorbidity burden.

Since the observations have a clustered structure defined by the geographical region, we define the region by $k \in \{1, \dots , K\}$ and the observation within the region as $l \in \{1, \dots , n_k\}$ such that $\sum _{k = 1}^{K} n_k = N$ with *K* being the total number of regions (i.e., $K=19$). The ordered beta regression [50] with random effect is estimated, where we assume the following distribution for $\textbf{y}$:

3 $$\begin{aligned} \begin{aligned} \Pr (y_{lk} = 0) =&1 - \text {logit}^{-1}(X_{lk} \varvec{\beta }_0 - k_1) \\ \Pr (y_{lk} = 1) =&\text {logit}^{-1}(X_{lk} \varvec{\beta } - k_2 + u_k) \\ \Pr (0< y_{lk} < 1) =&[\text {logit}^{-1}(X_{lk} \varvec{\beta } - k_1 + u_k) -\text {logit}^{-1}(X_{lk} \varvec{\beta } - k_2 + u_k) ]\\&\text {Beta} (\text {logit}^{-1}(X_{lk} \varvec{\beta } + u_k), \psi ) \end{aligned} \end{aligned}$$

where $\text {Beta}(\text {logit}^{-1}(X_{lk} \varvec{\beta } + u_k), \psi )$ is the Beta distribution with mean parameter $\text {logit}^{-1}(X_{lk} \varvec{\beta } + u_k)$ and precision parameter $\psi$.

The fixed covariates, represented by $\textbf{X}$, include socio-demographic and economic covariates, i.e., age, sex, education level, economic status, year, and interaction term between age and sex (described in Section "Italian surveillance system PASSI data"). These covariates influence the probability of each outcome (zero, one, or partial multimorbidity) within the three possible states. Focusing on the probability to be $\textbf{y} \in (0,1]$, we insert a regional random effect $u_k \sim \mathcal {N}(0, \sigma ^2_{u})$ along with the fixed effects defined by $\textbf{X}\varvec{\beta }$.

We reiterate here our decision to include random effects by region, as there is substantial evidence of regional and even sub-regional variability in health outcomes and health behaviors across Italy (see, for example, [17]). These differences are partly attributable to disparities in healthcare infrastructure and diagnostic capacity. For instance, the higher incidence of certain conditions, such as cancer, in northern regions may reflect more effective and widespread diagnostic procedures. Moreover, health-related behaviors vary geographically: alcohol consumption is more prevalent in north regions [51], whereas smoking habits are more common in the southern areas [52]. Ignoring such regional heterogeneity would obscure meaningful patterns in the data, resulting in national-level estimates that may significantly underestimate local differences and dynamics in multimorbidity.

The scalar parameters $k_1$ and $k_2$ are ordered cutpoints, i.e., $k_1 < k_2$, that permit relating the three probabilities [50]. So, the probabilities are no longer exchangeable as in a basic zero-one inflated model. Kubinec [50] proposed a Bayesian estimation approach using an induced Dirichlet prior over the set of cutpoints $\{k_1, k_2\}$. However, we rely on the glmmTMB R package [53], which uses a maximum likelihood-based optimization, assigning $k_1 = -1$ and $k_2 = 1$ as starting values. Model parameters are optimized using a combination of the nlminb optimizer and the template model builder [54] framework, which ensures computational efficiency even with large datasets.

### Results

**Fig. 4:**
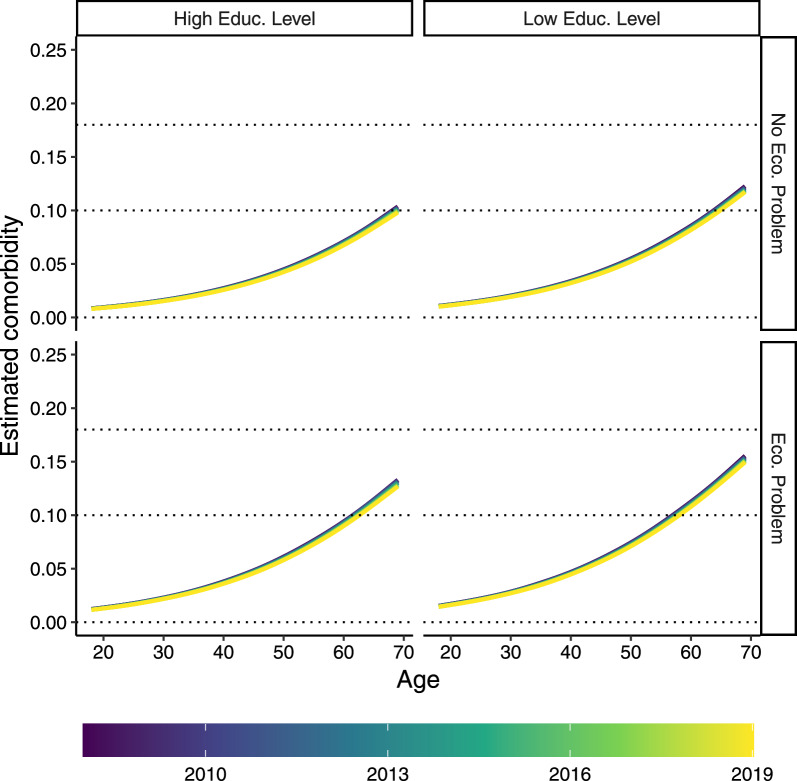
Marginal estimates of $\textbf{y}$ at the population level across age for the female subpopulation within each group categorized by levels of education and economic status. Colors denote the survey year from 2008 to 2019

**Fig. 5:**
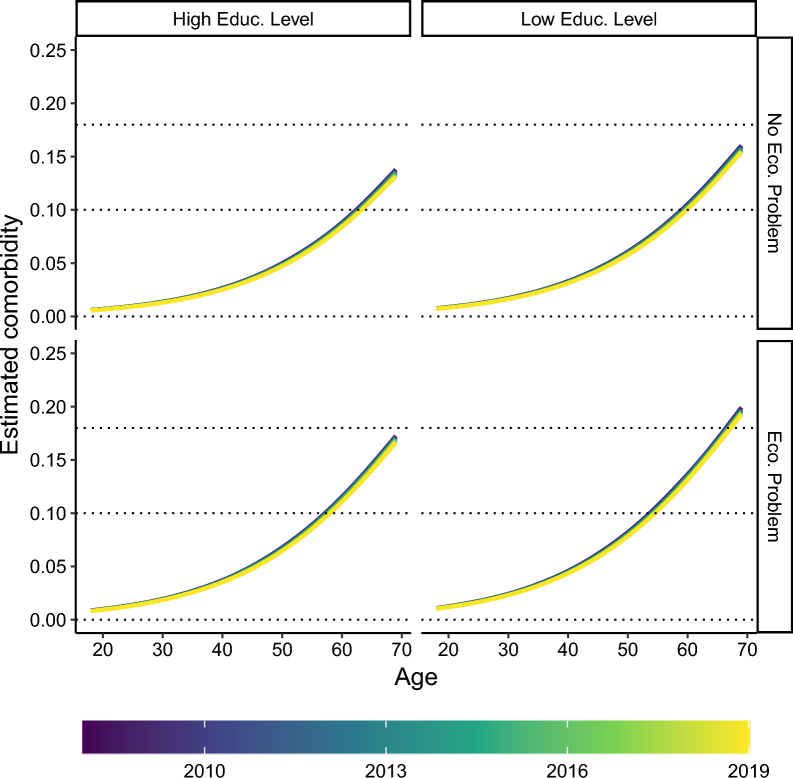
Marginal estimates of $\textbf{y}$ at the population level across age for the male subpopulation within each group categorized by levels of education and economic status. Colors denote the survey year from 2008 to 2019

Figures 4 and 5 present the population-level estimates of the multimorbidity index ($\textbf{y}$) across subpopulations stratified by educational attainment and economic status for female and male respondents, respectively. These estimates are obtained by combining the zero-inflated and conditional components of the model. Following the glmmTMB convention, “population-level” estimates refer to values computed by setting all random effects to zero, providing an approximation of the expected outcome for an average individual within each subgroup. The estimate for the *lk*-th observation is given by:

4 $$\begin{aligned} \hat{y}_{lk} = (1 - \hat{\gamma }_{lk}) \cdot \hat{\mu }_{lk} \end{aligned}$$

where $\hat{\gamma }_{lk} = \widehat{\Pr }(y_{lk} = 0)$ is the estimated probability of a zero outcome for the *lk*-th observation and $\hat{\mu }_{lk} = \widehat{\mathbb {E}}[y_{lk} \mid 0 < y_{lk} \le 1, X_{lk}]$ represents the expected value of the multimorbidity index with non-zero outcomes, conditional on the covariates $X_{lk}$. This formulation reflects the combined contribution of the zero-inflated and conditional components of the model. Although this approximation is standard in the glmmTMB framework, a more precise marginal estimate could be obtained by integrating over the distribution of the random effects, which is computationally more intensive.

**Table 2:** Summary of the mixed-mixture model defined in Equation (3), i.e., estimated coefficients, related standard errors, statistical tests for the null hypothesis of no effect, and related *p*-values

**Conditional model**	**Estimate**	**Std. Error**	*t* **value**	$\Pr (>|t|)$
(Intercept)	2.556	1.262	2.025	0.043
Sex (Male)	−0.089	0.019	−4.614	< 0.0001
Age	0.007	0.0003	28.011	< 0.0001
Economic Problem (No)	−0.065	0.005	−14.127	< 0.0001
Educational Level (High)	−0.075	0.005	−16.17	< 0.0001
Year	−0.002	0.001	−2.995	0.003
Sex (Male):Age	0.004	0.0003	10.535	< 0.0001

**Zero-inflation model**	**Estimate**	**Std. Error**	*t* **value**	$\Pr (>|t|)$
(Intercept)	−5.665	2.657	−2.128	0.033
Sex (Male)	0.558	0.039	14.166	< 0.0001
Age	−0.051	0.001	−99.493	< 0.0001
Economic Problem (No)	0.310	0.009	33.275	< 0.0001
Educational Level (High)	0.183	0.010	19.106	< 0.0001
Year	0.005	0.001	3.737	< 0.0001
Sex (Male):Age	−0.012	0.001	−16.278	< 0.0001

The top table shows the results regarding the conditional model (which predicts $y$ given that it is non-zero), while the bottom one presents the results of the zero-inflation model (which models the probability of the outcome being zero)

Looking at Fig. 5 and 4, we can note that males have greater levels of multimorbidity index than females in older ages, while the situation reverses in younger ages. Table 2 reveals a significant interaction between sex and age at the regional level, suggesting a possible crossover effect similar to that observed by Pastore and coauthors [7] at the population level.

Favorable social conditions, characterized by the absence of economic difficulties and a high level of education, were associated with a reduction in multimorbidity levels, as evidenced by the works of Pastore et al. [7] and Andreella and coauthors [29]. Specifically, focusing on the first year of the survey (2008), our model predicts a marginal multimorbidity level of 0.14 for males and 0.105 for females at age 69 under favorable social circumstances. In contrast, when examining the most vulnerable subpopulation, characterized by low educational attainment and economic challenges, the marginal predicted multimorbidity levels are higher, reaching 0.2 for males and 0.156 for females, again in 2008 at age 69. The literature indicates that these statements support the existence of disparities in multimorbidity across different socioeconomic classes [55, 56]. In contrast, encouragingly, the phenomenon of the compression of morbidity is observed not only in socially advantaged groups but also in high-risk populations and those facing social disadvantages.

Comparing multimorbidity values across socioeconomic classes, we observe a similar pattern of disparities when focusing on the last year of the survey, 2019. For the most advantaged population, the model predicts a marginal multimorbidity level of 0.133 for males and 0.1 for females. These values increase by 0.009 points for males and 0.008 for females in the most vulnerable population. This suggests that compression occurs across all subpopulations; however, in 2019, the multimorbidity level in the most vulnerable group remains higher than those in the most advantaged group in 2008. This trend evokes Zeno’s paradox of Achilles and the tortoise: while multimorbidity decreases, the reduction rate remains insufficient to equalize levels across all subpopulations.

**Fig. 6:**
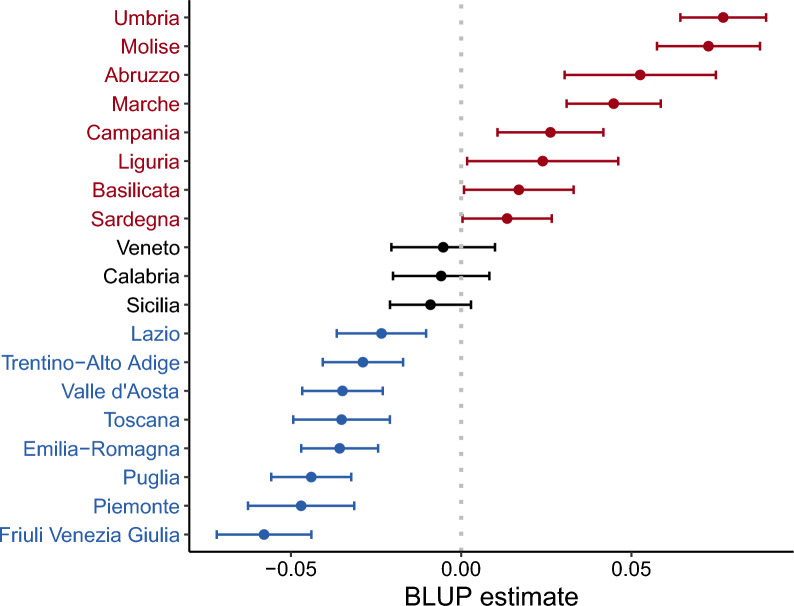
Best linear unbiased prediction (BLUP)

**Fig. 7:**
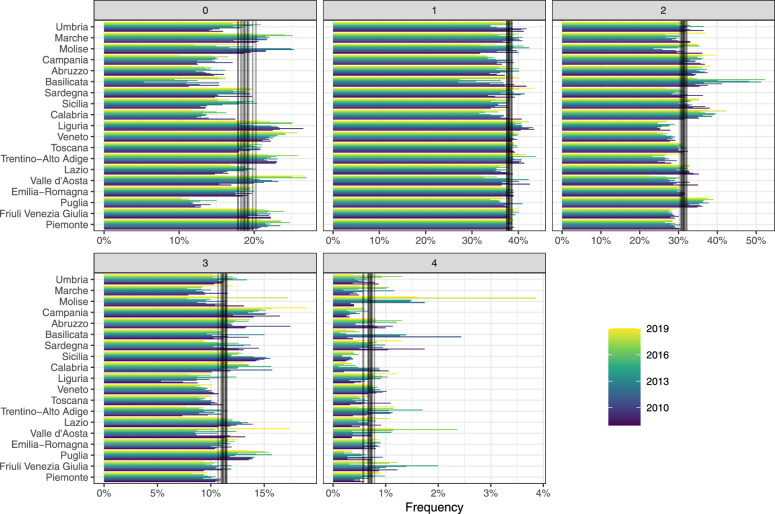
The conditional frequency is displayed for each year and region. Each panel represents a risk class defined by the cumulative count of four risky behaviors: smoking, alcohol consumption, poor diet, and physical inactivity, with values coded as 1 if the behavior is present and 0 if not. The vertical black lines represent the national averages for each year

Finally, Figure 6 presents each Italian region’s Best Linear Unbiased Predictions (BLUP). Notably, the data highlights that the Umbria region exhibits the most challenging scenario in terms of multimorbidity. Overall, the southern regions demonstrate a higher level of multimorbidity. The results are not surprising since these regions present a worse profile in terms of risk factor behaviors, as can be noted in Figure 7. We represent the conditional frequency by survey year and region; that is, for any given year and region, the sum of values across the five panels equals 1. Each panel corresponds to a sub-population with a specific profile of risky behaviors. The behavioral risk factors analyzed include smoking, alcohol consumption, poor diet, and physical inactivity, coded as 1 if the individual exhibits this behavior and 0 otherwise. Both current smokers and ex-smokers are considered to have a smoking risk factor, as smoking history details are unavailable in the PASSI data. In summary, a class risk score of 0 represents the optimal scenario with no risky behaviors, while a score of 4 indicates the highest risk, where all risky behaviors are present.

As a final analysis, we assess whether the negative effect of the survey year, i.e., the compression of multimorbidity, is acting and persists across sub-populations stratified by varying levels of risk factors, i.e., controlling for risk class. To this end, we fit the model specified in Equation (3) (i.e., ordered beta regression with random effects) separately for each of the five sub-populations, defined by risk class according to the cumulative count of four risky behaviors: smoking, alcohol consumption, poor diet, and physical inactivity. Here, we exclude the random effect for regions, focusing on the marginal rather than the conditional effect of the survey year. The results show a consistent negative year effect across sub-populations, with nearly equivalent effect magnitudes for class risks 0, 1, and 2. For class risks 3 and 4, the effect direction remains negative but is slightly less pronounced. These findings suggest that factors beyond changes in risky behavior prevalence, such as improvements in healthcare services or other latent factors, likely drive multimorbidity compression in Italy.

## Discussion

This study provides compelling evidence of multimorbidity compression in Italy, signifying a promising shift in public health outcomes. Our findings suggest that the onset of severe multimorbidities is increasingly delayed, and this delay is not solely attributed to reduced risk factors. Instead, it appears that broader improvements in healthcare accessibility and quality, combined with underlying socioeconomic factors, could be driving this phenomenon, as argued by Fries and coauthors [10, 10].

The use of a weighted latent trait model allowed us to capture the cumulative health burden of multiple chronic diseases as a single, continuous multimorbidity index. By integrating the GBD disability weights with PASSI data, this index highlights disparities across demographic and socioeconomic subpopulations. We observed that higher levels of multimorbidity persist among individuals with low educational attainment and economic hardship. Nevertheless, signs of compression–wherein multimorbidity is concentrated in later years of life–were present even within these more vulnerable subpopulations. This underscores the potential effectiveness of public health interventions that reach diverse demographic groups and address socioeconomic disparities. However, we have defined the presence of an “Achilles and the tortoise” effect, where the multimorbidity situation of the most vulnerable subpopulation is improving but not at a pace sufficient to reach the level of the most advantaged subpopulation. This finding aligns with recent Italian research (e.g., Pastore et al. (2023) [7] and Campostrini et al. (2014) [55]) as well as international studies such as Payne et al. [25] which report persistent disparities by education and race in the United States.

Further, our model accounted for geographical variability, revealing distinct regional patterns in multimorbidity consistent with the Italian findings of Stival et al. (2024) [17] and international evidence [16]. Southern regions, particularly Umbria, exhibit higher multimorbidity levels, likely influenced by higher prevalence of risk factors like smoking, poor diet, and physical inactivity. However, the consistent year-over-year decrease in multimorbidity across both high- and low-risk subpopulations indicates that the observed compression is influenced by factors beyond individual health behaviors alone. Our analysis suggests that multimorbidity compression is likely supported by multiple structural improvements within Italy’s healthcare and public health systems. These findings encourage further investigation into regional disparities and the impact of targeted healthcare policies on multimorbidity trends. Additionally, the approach and findings presented here could be applied to other health surveillance systems, such as the BRFSS in the United States [30], to explore multimorbidity compression in different contexts and populations.

Despite these findings, an important limitation is the cross-sectional nature of the PASSI data, which constrains our ability to draw causal inferences about the drivers of multimorbidity compression. Longitudinal or cohort-based data would be essential for identifying specific causal pathways and validating whether health interventions, policy changes, or socioeconomic shifts are directly responsible for delaying multimorbidity onset. Thus, while our results suggest a positive shift in public health outcomes, further studies are needed to confirm these trends and examine potential causal factors in depth. Another limitation is that this analysis does not account for life expectancy directly; instead, compression is inferred from the negative effect of survey year in the regression model rather than through a comparison of life expectancy with disease onset. However, we believe that measuring multimorbidity across several years is a valuable first step toward understanding the direction of these trends. Finally, a limitation of the latent trait model adopted in this study is that it does not explicitly account for potential interactions between specific symptom categories. The model assumes that the contribution of each condition to the latent morbidity burden is additive, which may overlook synergistic effects between co-occurring diseases. While this simplification facilitates interpretation and estimation, it may lead to an underestimation of the impact of certain comorbidity profiles. Future extensions could explore multidimensional or interaction-based frameworks to capture such complex dependencies better. Again, another limitation of this study is the restricted number of chronic conditions available in the PASSI dataset. Only five major non-communicable diseases (diabetes, chronic kidney disease, respiratory disease, heart disease, and cancer) are recorded. While these conditions are highly prevalent and represent key contributors to morbidity and premature mortality, they may not capture the full spectrum of multimorbidity in the population. Nevertheless, they serve as meaningful proxies for severe chronic morbidity, and their joint modeling allows us to estimate underlying trends consistent with the morbidity compression hypothesis. Future research could benefit from datasets encompassing a broader range of health conditions.

Our findings show that the Italian public health system needs important changes to reduce health differences between regions and social groups. Since multimorbidity varies a lot by region, healthcare plans should be made to fit local needs, with stronger primary care and better prevention services where they are needed most. Social policies should also focus on the main causes of poor health, especially education and economic difficulties, which our results show are closely connected to multimorbidity. Supporting community programs and making sure everyone can get good care will be key to helping people age healthily and reduce unnecessary pressure on hospitals and clinics.

In conclusion, this study’s evidence of multimorbidity compression is a positive sign, showing potential for better health outcomes across Italy. However, health inequalities between regions and social groups remain a concern. Future research should focus on healthcare and social policies that not only support this positive trend but also reduce these inequalities. This will help create a clear plan to manage multimorbidity in aging populations both in Italy and around the world.

## Additional files

### Unweighted latent trait model

**Fig. 8:**
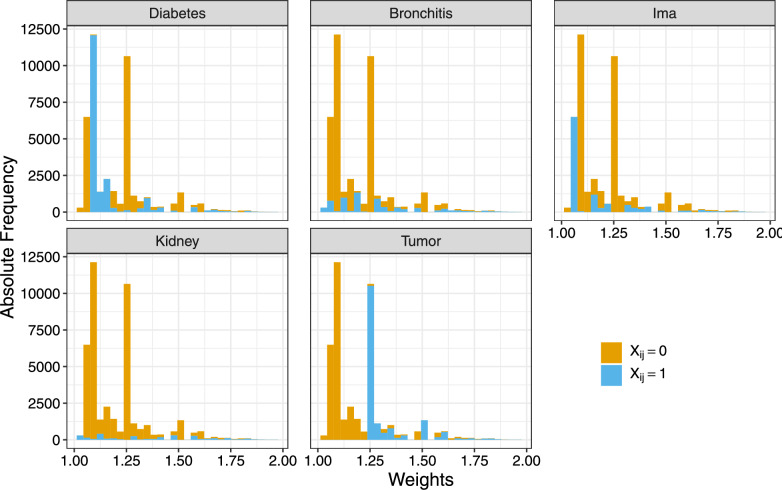
Frequency distribution of sampling weights greater/equals 1, differentiated by the presence (blue) and absence (orange) of each disease

Considering the estimated values of $\mathbf {\beta }_0$ and $\mathbf {\beta }_1$ under the unweighted latent trait model shown in Table 3, we observe that incorporating disability weights into the estimation process increases the apparent prevalence of tumor disease, indicated by a larger $\hat{\mathbf {\beta }}_0$. This is likely due to the relatively high disability weights associated with tumors, reflecting their substantial impact, as illustrated in Fig. 8. Fig. 8 presents the sampling weights applied in the LTM estimation, as defined in Equation (2), for cases where $X_{ij}$ equals 0 (no disease *i* reported) and 1 (disease *i* reported). For respondents reporting a tumor, the high disability weight directly influences the sampling weight, thus affecting the disease prevalence estimation.

**Table 3:** Estimate values of the unweighted latent trait model’s parameters

**Disease**	$-\hat{\varvec{\beta }}_0$	$\hat{\varvec{\beta }}_1$
**Diabetes**	3.745	1.332
**Kidney Failure**	6.148	1.979
**Respiratory**	2.911	0.909
**Heart**	4.050	1.613
**Tumor**	3.516	0.864

## Data Availability

No datasets were generated or analysed during the current study.
